# Effects of lifestyle physical activity on perceived symptoms and physical function in adults with fibromyalgia: results of a randomized trial

**DOI:** 10.1186/ar2967

**Published:** 2010-03-30

**Authors:** Kevin R Fontaine, Lora Conn, Daniel J Clauw

**Affiliations:** 1 Division of Rheumatology, Johns Hopkins University School of Medicine, 5200 Eastern Avenue, Baltimore, MD 21224, USA; 2 Departments of Anesthesiology and Medicine (Rheumatology), University of Michigan, Chronic Pain & Fatigue Research Center, 24 Frank Lloyd Wright Lobby M, Ann Arbor, MI 48106, USA

## Abstract

**Introduction:**

Although exercise is therapeutic for adults with fibromyalgia (FM), its symptoms often create obstacles that discourage exercise. We evaluated the effects of accumulating at least 30 minutes of self-selected lifestyle physical activity (LPA) on perceived physical function, pain, fatigue, body mass index, depression, tenderness, and the six-minute walk test in adults with FM.

**Methods:**

Eighty-four minimally active adults with FM were randomized to either LPA or a FM education control (FME) group. LPA participants worked toward accumulating 30 minutes of self-selected moderate-intensity LPA, five to seven days per week, while the FME participants received information and support.

**Results:**

Seventy-three of the 84 participants (87%) completed the 12-week trial. The LPA group increased their average daily steps by 54%. Compared to FME, the LPA group reported significantly less perceived functional deficits (*P *= .032) and less pain (*P *= .006). There were no differences between the groups on the six-minute walk test (*P *= .067), fatigue, depression, body mass index, or tenderness.

**Conclusions:**

Accumulating 30 minutes of LPA throughout the day produces clinically relevant changes in perceived physical function and pain in previously minimally active adults with FM.

**Trial Registration:**

clinicaltrials.gov NCT00383084

## Introduction

Fibromyalgia (FM) is a chronic, multidimensional disorder characterized by persistent, widespread body pain and tenderness [1]. FM is estimated to occur in 2% of the U.S. general population, affecting about eight times more women than men [2,3]. Symptoms associated with FM include body pain, fatigue, sleep disruption, headache, memory or concentration problems, mood disturbances, and irritable bowel syndrome [4]. FM often substantially hampers day-to-day functioning and is a primary cause of disability [5].

Even with the recent Food and Drug Administration approval of medications to treat FM, pharmacotherapy generally produces modest and inconsistent benefits on symptoms, functioning, and quality of life [6]. As such, nonpharmacologic treatments, such as exercise and cognitive-behavioral interventions, are recommended to assist people with FM to better manage the array of symptoms and functional deficits [6]. Although exercise has been shown to be beneficial [for example, [7]], the symptoms of FM often create obstacles that deter many from exercising consistently enough to derive benefits [8]. Thus, finding new ways to promote increased physical activity in persons with FM that can be sustained overtime is important.

One promising approach is to ask people with FM to increase their lifestyle physical activity (LPA). LPA involves working toward meeting the U.S. Surgeon General's 1996 Physical Activity Recommendations of accumulating at least 30 minutes, above one's usual activity, of moderate-intensity physical activity five to seven days a week by integrating short bouts of activity into the day, such as increasing the amount of walking, performing more yard work, using the stairs and so on [9-11]. Although it is unclear whether a continuous 30 minute bout of physical activity is superior to accumulating smaller (10- to 15-minute) bouts of activity with regard to health outcomes, asking people with FM to accumulate small bouts of physical activity throughout the day, as opposed to being active for 30 consecutive minutes, might be less taxing and therefore easier to initiate and sustain over time. In a pilot study [12], we found that small bouts of LPA promoted a 70% increase in physical activity in FM. However, in that small study LPA did not produce significant benefits on pain, fatigue, or perceived physical function compared to controls.

As part of an ongoing randomized trial designed to investigate the effects of LPA on ambulatory reports of physical activity, pain and fatigue, as well as measures of fitness, pain threshold and pain tolerance, we also collected questionnaire-based data on these variables. This paper presents the results on questionnaire-based assessments of perceived physical function, pain, fatigue and depression, as well as tenderness and aerobic endurance after 12 weeks of LPA in minimally active adults with FM.

## Materials and methods

### Participants

Participants were 92 adults (88 women and 4 men) aged 18 years or older who met American College of Rheumatology diagnostic criteria for FM [13]. The mean (SD) age of participants was 47.7 ± 10.7 years and 80% were white. The mean duration of FM was 7.5 ± 6.2 years. At enrollment, participants were not meeting the US Surgeon General's 1996 recommendation for physical activity [11] for the previous six months (that is, not engaging in either moderate-intensity physical activity for ≥ 30 minutes on ≥ five days per week or vigorous physical activity ≥ three times per week for ≥ 20 minutes each time during the previous month). Persons with acute or chronic medical conditions that could preclude active participation (for example, cancer, coronary artery disease) were excluded from the trial. We also excluded those who intended to change medications that might affect mood, those who intended to seek professional treatment for anxiety or depression during the study period, and those who were unwilling to make the required time commitment.

Participants were recruited from the Johns Hopkins Arthritis Center, affiliated Johns Hopkins Rheumatology clinics, by advertisements in the Arthritis Foundation Maryland Chapter newsletter, newspaper advertisements, and via clinical trial recruitment websites, including clinicaltrials.gov. All participants completed baseline testing which included a series of questionnaires, a tender point examination, and a six-minute walk test. At baseline, participants also wore a waist-mounted pedometer (AccuSplit Eagle 1020, Livermore, CA, USA) for seven days (recalibrating it each morning) and recorded their daily step count. These data were used to calculate the mean steps per day as an estimate of physical activity. This study was approved by the Institutional Review Board of Johns Hopkins University School of Medicine, and all participants gave written informed consent.

### Study procedures

Participants were randomized via a coin flip at a 1:1 allocation ratio to each of the two groups. The group meetings for LPA and FME were held on different days to avoid contact between participants assigned to the different conditions. The interventions did not replace usual medical care and the participants had comparable durations of contact time with study staff (Table 1 summarizes the LPA and FME conditions).

**Table 1:** Description of lifestyle physical activity (LPA) and fibromyalgia education (FME) protocols*

Component	LPA	FME
**Three, two-hour FM education and support meetings**	NO	YES
**Physical activity intervention delivered in six, one-hour meetings**	YES	NO
**Wear pedometer and keep a physical activity log**	YES	NO
**Prescribed physical activity**	YES	NO
**Approximately six hours of face-to-face contact time**	YES	YES
**Topics Covered During the Meetings**		
LPA	FME	
"Physical Activity & FM" Described FM (symptoms, diagnosis, treatment)/benefits of physical activity/demonstrated moderate-intensity LE/prescribed LE and self-monitoring/identified and addressed barriers to physical activity *(Sessions 1 & 2)*	"FM: What is it and how is it diagnosed?" Presented general information on the symptoms of FM and how it is diagnosed; Discussion and social gathering (Session 1)	
"How to Keep Moving" Discussed progress, effect on symptoms, goal setting, problem solving, importance of self-monitoring, provided feedback, and troubleshooting *(Sessions 3 & 4)*	"What causes FM?" Presented the latest information on the causes and consequences of FM; Discussion and social gathering (Session 2)	
"Now It's Up To You" Planned for setbacks & developed strategies to overcome them, set long-term goals, self-monitoring over the long-term *(Sessions 5 & 6)*	"Treating FM" Discussed of medical and non-medical approaches, including exercise, to treating FM; Discussion and social gathering (Session 3)	
LPA Accumulate ≥ 30 minutes of self-selected physical activity five to seven days per week	FME Did not alter their characteristic level of physical activity	

*FM, fibromyalgia; FME = fibromyalgia education; LPA, lifestyle physical activity

### Lifestyle physical activity (LPA)

Participants assigned to LPA attended six, 60-minute group sessions over 12 weeks. Delivered by one of the authors (KRF), the LPA protocol was identical to the one developed for our pilot study [12] and was loosely based on *Active Living Every Day*, a cognitive-behavioral physical activity promotion program developed by Dr. Steven Blair and colleagues at the Cooper Aerobics Center [14]. The LPA protocol addressed FM-specific challenges to becoming more physically active (that is, dealing with pain and fatigue, fear that physical activity will promote a flare) and discussed how LPA successfully addresses them. The goal of the LPA intervention was to increase moderate-intensity physical activity by helping participants find ways to accumulate short bouts of physical activity throughout the day. Participants were asked to gradually work their way up to meeting the Surgeon General's 1996 recommendation of accumulating 30 minutes, above usual activity, of moderate-intensity LPA five to seven days each week.

At the first session, participants were taught how to perform their LPA at moderate-intensity (that is, you will be breathing heavily but not so heavily that you could not hold a conversation). They were also prescribed 15 minutes, above usual level, of accumulated moderate-intensity LPA five to seven days a week, and asked to increase the daily duration of LPA by five minutes each week. The five-minute increase in the daily duration of LPA was based on findings from our pilot study [12] and was well-tolerated by the majority of participants. Thus, by Week 5, most participants were accumulating 30 minutes, above their usual level, of LPA five to seven days a week. Participants were free to accumulate more than 30 minutes of LPA five to seven days per week, if desired.

During subsequent sessions participants were taught self-monitoring of LPA, goal setting, dealing with symptom flares, problem solving strategies to overcome barriers to being more physically active, as well as instruction in finding new ways to integrate short bouts of LPA into their daily lives. Feedback focused on whether participants achieved the prescribed level of LPA, as well as the LPA's influence on symptoms.

Participants wore the waist-mounted pedometers to record their steps each day (that is, as an assessment of adherence to LPA). Participants were shown how to use the pedometer, where to place it, and how to record their steps on a step diary form. At the end of each day they recorded their steps on a diary form and *zeroed *their pedometer so they could record their steps for the next day. They also kept a diary that categorized the types of LPA's they engaged in (for example, garden/outdoor activity, household activity, leisure activity). The step count data and diary entries were collected at each intervention session.

### Fibromyalgia education (FME)

Participants assigned to the FME group met monthly for three months. FME was a minimal intervention with each session divided into three components: (1) education (45 to 60 minutes), (2) question and answer (20 to 30 minutes), and (3) social support (20 to 30 minutes). Conducted by an experienced FM support group facilitator, these 90- to 120-minute sessions presented information on the symptoms, diagnosis, and treatment of FM. The rationale for FME was to provide education and to control for the effects of being enrolled in a clinical trial and receiving increased attention and social support. Moreover, by providing a minimal intervention, as opposed to a standard care control, we anticipated enhancing retention. The final session of FME presented information on exercise and physical activity, but no specific recommendations or prescription concerning exercise was given. To avoid the possibility that wearing a pedometer would increase their physical activity, FME participants only wore one for the baseline and post-testing assessments.

### Outcomes measures

The following outcome measures were collected at baseline and after the 12-week intervention.

### Primary outcome

#### Perceived physical function

Perceived physical function was assessed using the Fibromyalgia Impact Questionnaire (FIQ) total score. The FIQ is a well-validated 10-item questionnaire that measures aspects of physical functioning in patients with FM [15]. The FIQ is scored so that higher scores are indicative of poorer functioning. Test-retest reliability ranged from .56 to .95 and construct validity relative to tender points was acceptable (rs = approximately .56) [15].

### Secondary outcomes

#### Pain

Pain was assessed using a 100 mm Visual Analogue Scale (VAS) where participants rated their current level of pain, ranging from 0 (no pain) to 100 (worse pain imaginable).

#### Fatigue

The Fatigue Severity Scale (FSS) [16] was used to assess the current level of fatigue. The FSS is a nine-item questionnaire, answered on a seven-point scale, ranging from *strongly agree *to *strongly disagree*. The FSS has good internal consistency (Cronbach's alpha = .81) and correlates with VAS fatigue measures (r = .68) [16].

#### Depression

Depression was assessed using the Center for Epidemiologic Studies Depression Scale (CES-D) [17]. The CES-D contains 20-items rated on a four-point Likert scale ranging from 0 (rarely or none of the time) to 3 (most or all of the time), and measures symptoms during the past week. The CES-D is a widely used measure of depressive symptoms and has acceptable internal consistency (.84 to .90) and validity (r = .56 with clinical rating of depression severity) [17].

#### Tenderness

A digital tender point examination, at the 18 sites specified in the American College of Rheumatology FM classification criteria [13], was completed at baseline and after the intervention. Tender point counts are moderately reliable in classifying the tenderness associated with FM (kappa = .75) and inter-rater agreement on the presence of tenderness through digital examination is .51 [13].

#### Body mass index (BMI: kg/m^2^)

Weight and height were recorded at each assessment and these variables were used to calculate BMI, an index of body weight adjusted for height.

#### Six-minute walk test

The six-minute walk [18] is a measure of aerobic endurance. For this test, participants walked as far they could in six minutes on a preselected course, with the distance walked recorded. The reliability of the six-minute walk test is excellent (r = .91) and it correlates with the FIQ (r = -.49) and is sensitive to change due to exercise in distance walked (+78 m), and VO_2 _(+1.8 ml/kg/min) [18]. The six-minute walk test was measured at baseline and at post-testing. We expressed the results as meters per second, an index of gait speed.

### Sample size and data analysis

Thirty-five adults with FM per group were projected to provide a power of 80% to detect a clinically significant 20% difference between the groups on the FIQ score. Ninety-two participants were enrolled to allow for a 25% post-randomization drop out rate.

Baseline data between the two groups were compared using t-tests or Chi Square tests. Changes in perceived physical function, depression, pain, tenderness, fatigue, BMI, and the six-minute walk test were compared between the LPA and FME groups using between-subjects t-tests. Because there was a significant difference between the LPA and FME groups on self-reported duration of FM (see Table 2), as a sensitivity analysis, we adjusted scores of the outcome measures for the duration of FM and replicated the analyses. We also used regression techniques to adjust the outcome measures on the basis of whether or not the participant reported any change in their ongoing FM treatments, either pharmacologic or non-pharmacologic (0 = no change, 1 = change) during the trial. Because the results did not differ as a function of these adjustments, we present the results for the unadjusted outcome variables. Although data from all subjects were analyzed regardless of whether those subjects complied with or remained in treatment, participants with missing data on a particular variable were excluded from that particular analysis. We also performed an analysis among participants who completed the 12-week trial (*completers only*). Cohen's *d *effects size estimates [19] were calculated for each difference on the outcome measures between LPA and FME. Analyses were performed using SPSS software, Version 16. Two-tailed *P *values of < 0.05 were used to denote statistical significance.

**Table 2:** Baseline characteristics of the randomized participants*

Characteristic	Lifestyle Physical Activity (LPA)	Fibromyalgia Education (FME)	*P *value
N (%) of participants	46 (55)	38 (45)	
Age, years	46.4 ± 11.6	49.0 ± 10.2	0.287
Female, N (%)	43 (94)	38 (100)	0.248
Self-reported race, N (%)			0.789
White	36 (78)	31 (82)	
Non-White	10 (22)	7 (18)	
Marital status, N (%)			0.519
Married or cohabitating	24 (52)	24 (63)	
Widowed, divorced, or separated	12 (27)	11 (29)	
Single	10 (22)	3 (8)	
Educational level, N (%)			0.454
Postgraduate	9 (20)	5 (13)	
College graduate	16 (34)	11 (32)	
Some college	11 (24)	13 (34)	
High school	10 (22)	8 (21)	
Employment status, N (%)			0.923
Employed	20 (43)	18 (47)	
Unemployed or Disabled	11 (24)	9 (24)	
Retired or Other	15 (33)	11 (29)	
Years since diagnosis^a^	5.9 ± 5.1	9.6 ± 6.8	0.007
Steps per day	3,788 ± 2,135	3,071 ± 1,810	0.139
Taking FM medications, N (%)^a^	31 (82)	40 (87)	0.498
Taking other medications, N (%)^a^	33 (87)	44 (96)	0.146

* Data for categorical variables are presented as N' and percentages; data for continuous variables are presented as means ± standard deviation.

^a ^Obtained from self-report.

FME, fibromyalgia education; LPA, lifestyle physical activity

## Results

**Figure 1:**
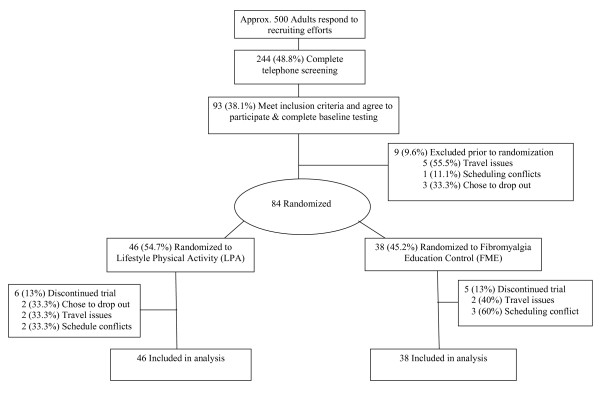
**Participant flow**.

Nine participants withdrew after baseline testing but prior to randomization (see Figure 1). We randomized 46 participants into the LPA intervention and 38 into the FME group in five separate cohorts of approximately 8 to 10 per cohort at six-month intervals. (Because the FME facilitator was unavailable, one smaller cohort (N = 4) was comprised of only LPA participants). Selected baseline characteristics of the 84 participants are shown in Table 2. With the exception of duration of FM, the two groups were comparable on age, race, education, employment status, BMI, and the use of medications for FM or for other medical conditions.

Seventy-three of the 84 participants (87%) completed the 12-week intervention and post-testing. Drop outs were unrelated to randomized treatment assignment (*P *= .988) and there were no significant differences on any baseline variables between those who dropped out and those who completed post-testing. There was also no difference in the mean percentage of meetings attended by those randomized to the FME (77%) and LPA (72%) groups (*P *= .542).

**Figure 2:**
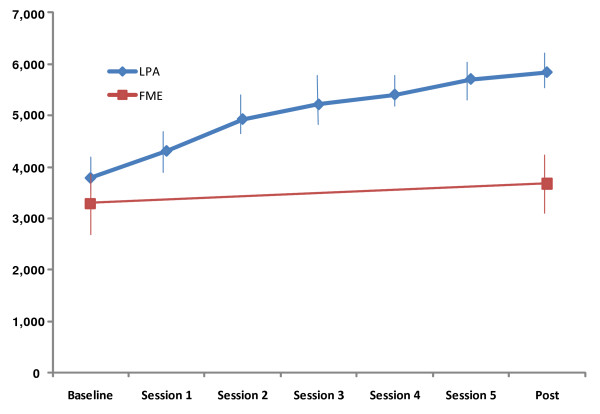
**Average steps per day (with 95% confidence interval) for the study groups**.

As shown in Figure 2, the LPA group significantly increased the mean number of daily steps from 3,788 ± 2,135 at baseline to 5,837 ± 1,770 at the final intervention session (*P *= .001). This represents a 54% increase in the mean number of daily steps over the course of the 12-week intervention. Although walking was the most common form of LPA, other popular forms of LPA included garden/outdoor activity (for example, mowing the lawn, planting flowers, pulling weeds); household activity (for example, cleaning out a closet, vacuuming, doing laundry); and sports activity (for example, cycling, swimming, field hockey).

At baseline, there were no significant differences between the LPA and FME groups on FIQ, pain, fatigue, depression, number of tender points, BMI, and six-minute walk distance (see Table 3). At post-testing, compared to the FME group, the LPA group reported significant reductions in the FIQ score (*P *= .032; Cohen's *d *= .53) and in pain (*P *= .006; Cohen's *d *= .67). The difference between the LPA and FME groups on the six-minute walk test approached significance (*P *= .067; Cohen's *d *= .53). There were no significant differences between the groups on BMI, fatigue, depression, or the number of tender points. The results (data not shown) were not materially altered when the analysis was restricted to only participants who completed the entire 12-week trial (that is, *completers only *analysis).

**Table 3:** Differences between lifestyle physical activity (LPA) and fibromyalgia education (FME) groups on the primary and secondary study measures^a^

Variable		Mean ± SD		Mean difference between groups at baseline and at 12-weeks (95% CI)	*P *Value	Cohen's *d*
	
	N	LPA	FME			
FM impact questionnaire						
Baseline	84	67.5 ± 12.0	69.7 ± 13.4	2.2 (-3.3 to 7.8)	0.424	.17
Post intervention	73	56.7 ± 20.6	67.0 ± 18.6	10.2 (.91 to 19.6)	0.032	.53
Pain						
Baseline	84	54.6 ± 25.6	58.9 ± 25.0	4.3 (-7.2 to 14.9)	0.489	.17
Post intervention	73	46.3 ± 24.2	62.4 ± 24.5	16.1 (4.6 to 27.5)	0.006	.67
Fatigue severity scale						
Baseline	84	51.9 ± 9.3	52.3 ± 9.1	.4 (-3.6 to 4.4)	0.843	.04
Post intervention	73	50.6 ± 9.9	51.4 ± 10.1	.8 (-3.9 to 5.5)	0.727	.07
CES-D*						
Baseline	84	23.4 ± 8.6	24.0 ± 10	.6 (-3.8 to 4.5)	0.798	.06
Post intervention	73	21.6 ± 9.8	21.2 ± 11.3	.4 (-5.3 to 4.6)	0.888	.04
Number of tender points						
Baseline	84	16.2 ± 2.3	16.1 ± 3.2	.1 (-1.2 to 1.0)	0.979	.03
Post intervention	72	16.0 ± 2.3	16.8 ± 2.0	.8 (-.35 to 1.9)	0.172	.37
Body mass index (BMI)						
Baseline	82	31.4 ± 8.4	29.8 ± 6.2	1.6 (-4.7 to 1.7)	0.360	.22
Post intervention	60	31.0 ± 9.0	29.9 ± 6.2	1.1 (-5.3 to 2.9)	0.575	.14
Six-minute walk test, yd/sec						
Baseline	77	1.08 ± 0.15	1.08 ± 0.19	.0004 (-0.78 to 0.79)	0.991	0
Post intervention	62	1.24 ± 0.28	1.11 ± 0.20	1.21 (-0.25 to 0.008)	0.067	.53

* Center for Epidemiologic Studies Depression Scale; ^a ^between-subjects t-tests were used to derive P values; ^b^Cohen's *d *[19] effect size estimates (*d *= .20 (small effect); *d *= .50 (medium effect); *d *= .80 (large effect)).

FME, fibromyalgia education; LPA, lifestyle physical activity

## Discussion

The 12-week program, designed to help minimally active adults with FM increase their physical activity by working toward accumulating at least 30 minutes of self-selected moderate-intensity physical activity most days of the week, produced a 54% increase in the average number of steps taken per day. Compared to the FME control group, LPA participants significantly reduced their perceived functional deficits (that is, FIQ score) and pain. Moreover, compared to FME, the LPA participants had a greater improvement on the six-minute walk (expressed as gait speed), although this difference failed to reach statistical significance. The magnitude of the post-intervention differences, expressed as percent change from LPA to FME groups, were 18% for the FIQ score and 35% for the pain VAS score. When expressed as Cohen's *d *effect sizes these are indicative of medium-sized effects. Moreover, the change on the FIQ score exceeds the minimally clinically important difference of 14% recently identified [20], suggesting that increasing physical activity via LPA produces changes on perceived physical function that are of a relevant magnitude. On the other hand, the effect of LPA on the six-minute walk test was not statistically significant (although it produced a Cohen's *d *of .53). It is important to note that there was a smaller sample size available for this analysis which reduced statistical power.

In general our results are in accord with studies investigating the effects of exercise on people with FM [7,8,21,22]. Specifically, the majority of studies suggest that exercise can produce mild-to-moderate benefits on aerobic endurance, strength, functional status, and quality of life [7,23,24]. However, because the exercise interventions investigated vary so markedly in type (for example, water aerobics, traditional aerobics, T'ai Chi, strength training), frequency, intensity, and duration it is difficult to compare results across studies.

One thing seems clear from the FM exercise literature, people with FM have difficulty adhering to exercise. Indeed, in FM clinical exercise trials drop-out rates often nearly exceed 30% [for example, [8,24]] suggesting that developing exercise interventions that can be sustained is perhaps as important a goal as finding the particular interventions that produce optimal benefits.

The magnitude of the effects of LPA observed in this study on perceived physical function and pain were similar to those obtained in our smaller pilot study [12]. These effects were also generally consistent with other protocols that involve low-to-moderate intensity exercise, interventions that appear to produce the greatest level of compliance in people with FM [for example, [7,8,24]].

It is important to note that even though the LPA group increased their mean daily steps by 54%, it only moved them, as defined by the pedometer-determined physical activity classifications developed by Tudor-Locke and colleagues [25], from the sedentary (<5,000 steps/day) to the low active (5,000 to 7,499 steps/day) category. Indeed, the mean steps per day at post-testing among the LPA participants were comparable to the mean daily steps observed in patients with progressive neuromuscular disease, and are significantly lower than other special populations such as diabetics, patients undergoing breast cancer treatment, and those with joint replacements [26]. This suggests that, even with the initiation of LPA, people with FM progress only to a relatively low level of physical activity. It is important to note, however, that the trajectory of the mean step count continued to rise over the 12 weeks suggesting that, had the trial continued, their physical activity may have continued to increase. It may be that people with FM require more time to eventually reach physical activity recommendations compared to persons with other chronic conditions.

This study has limitations and strengths. First, to minimize attrition and control for the effects of increased attention, participants randomized to the FME group did receive a minimal intervention. Thus, we cannot determine how LPA compares with a traditional no treatment control group. Second, with the exception of BMI, the tender point count and six-minute walk test, the outcomes described herein were derived from self-report and may be influenced by a variety of factors, including those associated with enrollment in a clinical trial. Third, using pedometers to assess LPA is relatively crude and does not quantify other sorts of physical activities that participants may have engaged in such as cycling or water exercise. Fourth, we did not measure muscle strength during the trial so we are unable to determine whether LPA influences strength. Finally, we excluded persons with FM who had other co-morbid conditions such as uncontrolled hypertension or arthritis which may limit the generalizability of our findings. Strengths of this study include the randomized design, a relatively small drop-out rate (13%), the LPA group's adherence to standardized intervention protocol, and the relatively high rates of attendance to the group sessions.

## Conclusions

The results of this study suggest that promoting increased physical activity by asking persons with FM to accumulate short bouts of activity throughout the day can markedly increase the average number of steps taken per day and produces clinically relevant reductions in perceived functional deficits and pain. However, the LPA intervention only moved the participants from the sedentary to low physical activity category. This suggests that it is essential to encourage FM patients to increase the duration of their physical activity in ways that do not compromise their ability to sustain the increased level of activity over the intermediate- and long-term.

## Abbreviations

BMI: Body Mass Index; CES-D: Center for Epidemiologic Studies Depression Scale; FIQ: Fibromyalgia Impact Questionnaire; FM: fibromyalgia; FME: Fibromyalgia Education Control Group; FSS: Fatigue Severity Scale; LPA: lifestyle physical activity; SD: standard deviation; VAS: Visual Analogue Scale.

## Competing interests

Kevin R. Fontaine and Lora Conn declare that they have no competing interests. Daniel J. Clauw has acted as a consultant for Pfizer, Lilly, Forest Laboratories, Cypress Biosciences, Pierre Fabre, UCB, and Wyeth, and has received grant support from Pfizer, Cypress Bioscience, and Forest.

## Authors' contributions

KF conceived of the study, acquired the funding, participated in the design of the study, the delivery of the intervention, performed the statistical analysis, and drafted the manuscript. LC carried out the recruitment, enrollment, and data collection. DC participated in designing the study and assisted with the drafting of the manuscript. All authors read and approved the final manuscript.
